# Correlation Analysis Between *MTHFR C677T* Polymorphism and Uterine Fibroids: A Retrospective Cohort Study

**DOI:** 10.3389/fonc.2021.648794

**Published:** 2021-06-01

**Authors:** Jiahui Shen, Yanhui Jiang, Fengzhi Wu, Hui Chen, Qiujing Wu, Xiaoxiao Zang, Le Chen, Yong Chen, Qiwen Yuan

**Affiliations:** ^1^ Department of Obstetrics, The Fifth Affifiliated Hospital of Sun Yat-sen University, Zhuhai, China; ^2^ Department of Gynecology, The Fifth Affifiliated Hospital of Sun Yat-sen University, Zhuhai, China

**Keywords:** uterine fibroids, UF, Methylenetetrahydrofolate reductase, MTHFR, heritability

## Abstract

**Background:**

Uterine fibroids(UF) are the most common benign tumors in women, with high incidence and unknown causes. We aimed to explore the correlation between Methylenetetra-hydrofolate reductase (*MTHFR*) *C677T* polymorphism and UF.

**Methods:**

This is a retrospective cohort study. Data were collected from 2411 women detected for *MTHFR C677T* polymorphism in the Fifth Affiliated Hospital of Sun Yat-sen University from 2018 to 2020. B-ultrasound (BU) and the first page of medical records were used to analyze whether they had ever been diagnosed with UF. The collected data were analyzed. Using the chi-square test and regression analysis to explore the correlation, and the risk factors was screened by multifactor logistic regression analysis.

**Results:**

A total of 2411 pregnant women were in the *MTHFR C677T* polymorphism detection. Among them, 226(9.37%) were diagnosed as UF by BU or clinical diagnosis. The allele and genotype of *MTHFR C677T* were significantly different between the case and control group (p<0.05), and the distribution of the allele was following Hardy-Weinberg (H-W) equilibrium. Comparing with the wild-type (*C/C*), the mutant group (*C/T+T/T*) was more likely to form UF(OR,1.43;OR95%CI,1.07-1.89). After adjusting for confoundings, the heterozygous mutant (*C/T*) was more susceptible to UF than the wild-type (aOR,1.41;aOR95%CI,1.41-1.91). In the case group, BMI, gravidity and parity were not associated with the size and number of UF and the *MTHFR C677T* polymorphism (p>0.05). However, older maternal age was associated with the incidence of UF, especially the multiple UF (p<0.05).

**Conclusion:**

Our results found that *MTHFR C677T* polymorphism was associated with UF occurrence for the first time. This could imply that it may increase the risk of forming UF in women of gestational age.

## Introduction

Uterine fibroids (UF), originating from the Smooth muscle layer of the uterus and are composed of Smooth muscle cells (SMC) and connective tissue, are the most common benign tumour in gynecology with specific hereditary characteristics (1). They are highly prevalent, the incidence is as high as 70% and more common in women of childbearing age, and most of them are asymptomatic, and the rate of malignant degeneration is low. However, it often causes abnormal uterine bleeding, secondary anemia, pelvic organ pressure, infertility and pregnancy. It may even appear during pregnancy with severe complications such as red degeneration and is one of the main diseases leading to hysterectomy (2). With the development of ultrasound technology, the incidence of UF infertile women has increased from about 35% (clinically diagnosed only) to about 50% (ultrasono-graphy) (3). Moreover, the annual treatment cost is high that imposes a considerable burden on medical resources and poses a severe threat to women’s physical and mental health by the end of their reproductive years. With the opening of China’s two-child policy, more attention had paid to the impact of UF on women of childbearing age (4). It has a significant impact on the preparation, process and outcome of pregnancy on women of childbearing age that increasing the incidence of adverse pregnancy outcomes and maternal mortality to a certain extent. Therefore, it is crucial to identify the risk factors for UF and long-term quality of life in young women of childbearing age.

However, the widespread prevalence of the disease and the mechanisms involved in its growth are largely unknown, leading to slow progress in developing effective treatment options (5). Most previous studies have shown that UF are related to the level of sexual arousal in women and the number of hormone receptors expressed on the myometrium’s surface, but the cause and mechanism of action are still unclear that the treatment means are limited (6). Recently, an increasingly popular view suggested that UF may result from a consequence of a chronically active inflammatory immune system that secreted related inflammatory factors as mediators of sexual steroids and involved in UF’s formation and proliferation fibrosis and angiogenesis (7). Therefore, It is defined by the accumulation of excess extracellular matrix (ECM) components. Its occurrence may be related to the imbalance of the inflammatory process, oxidative stress (OS), and the differential expression of various growth factors involved in angiogenesis. At the same time, hormones may promote fibroids’ growth by activating fibroblasts, but the mechanism is not certain (8). Reactive oxygen species (ROS) is the main source of superoxide and subsequent oxidative stress. Folic acid (FA) is an antioxidant that can reduce the production of ROS (9). Recent studies have shown that FA supplementation can reverse the disease by reducing oxidative stress and fibrosis (10), and high homocysteine(Hcy) can up-regulate vascular endothelial growth factor(VEGF) promoting the occurrence and development of the disease.

*MTHFR* plays a key role in the enzymatic process in the folate metabolism pathway, and its principal function is to convert 5,10-methylenetetrahydrofolate to 5-methy- ltetrahydrofolate with biological functions, and then participate in DNA synthesis, modification and methylation (11). The *MTHFR* gene is composed of 11 exons and located on the short arm of chromosome1(1p36.3) (12). It is one of the most common mutations at position 677 in exon 4 that the cytosine (*C*) was replaced by the thymine (*T*) (13). Its 222nd amino acids in the protein alanine into valine and eventually leads to the decrease of *MTHFR* activity, the increase of Hcy level, and the decrease of DNA synthesis and methylation (14). Numerous epidemiological studies have shown that abnormal folate metabolism and high Hcy are resulting in abnormal DNA methylation, vascular endothelial cell injury and dysfunction of blood coagulation, and causing many systems many kinds of diseases, such as the tumors of gynecology, digestive system and nervous system, cardiovascular diseases and systemic diseases (15). At present, there are no relevant reports on UF and *MTHFR C677T* polymorphism, and the correlation between them remains unclear. *MTHFR C677T* polymorphism mostly leads to abnormal FA metabolism and increased Hcy level, while FA supplementation can significantly reduce the Hcy content in tissues (16). At the same time, studies have shown that UF has a specific genetic correlation (10), but there have been no reports on the *MTHFR C677T* polymorphism and UF’s occurrence and development, and its effect on the occurrence and development of UF is unknown.

The authors undertook a retrospective study to analyze the correlation between *MTHFR C677T* polymorphism and UF to explore UF’s pathogenesis factors and that will be a promising intervent approach for the prevention and treatment of UF.

## Materials and Methods

### Patient Enrollment

This study’s subjects were women aged 20-45 years of childbearing age who visited the Fifth Affiliated Hospital of Sun Yat-sen University and underwent the *MTHFR C677T* polymorphism detection between 2018 and 2020. Considering *MTHFR C677T* genotypes can differ depending on ethnicity and smoking incidence, and ethnicity or maternal smoking may be a confounding factors. Our study’s ethnicity is Han, and the women with a history of smoking was excluded. The first page of the medical record and B-ultrasound system were used to screen the diagnosis of UF. Relevant data obtained through the outpatient medical record system, examination system, ultrasonic examination system, and inpatient electronic medical record system, and these data were analyzed retrospectively. Subjects were separated into the case group and the control the control group based on the diagnosis of UF.

*MTHFR A1298C* and *C677T* mutations are associated with folate metabolism, however, it is common to detect *MTHFR C677T* polymorphism in our hospital. The number of cases detected by *MTHFR A1298C* polymorphism is still too small to be included in the study, so our study has not been included in the analysis.

Inclusion criteria were as follows: (1) All women are Han women, and women had detected the *MTHFR C677T* polymorphism in our hospital; (2) She had B-ultrasound examination in our hospital and indicated or not that she had uterine fibroids. (3) She had hospitalized in our hospital or another hospital for UF.

Exclusion criteria: (1) women who had the detection of the *MTHFR A1298C* polymorphism but without the detection of *MTHFR C677T* polymorphism, (2) women who had the detection of the *MTHFR C677T* polymorphism in our hospital, but they never had the B-ultrasound examination in our hospital or outside the hospital. (3) other minority women. (4) women with a history of smoking. (5) women who had the detection of the *MTHFR A1298C* polymorphism.

The Review Committee of the Fifth Affiliated Hospital of Sun Yat-sen University approved the study. Considering that our study was retrospective, no informed consent is required.

### Genotyping Methods

The *MTHFR C677T* polymorphism was detected in the Molecular Biology Laboratory of the Fifth Affiliated Hospital of Sun Yat-sen University. And the PCR reaction solution of the *MTHFR C677T* Polymorphism Detection Kit contains (Wuhan Youzhiyou, China, batch number: C12Q1/68): PCR buffer, dNIPS, specific probe (*MTHFR 677C*:5’-FAM-GTGTCTGCGGGAGCCG-NFQ-MGB-3’ and *MTHFR 677T*: 5’-VIC-GTGTCTGCGGGAGTCG-NFQ-MGB-3’), internal standard primers(F sequence of forward internal standard primer:5’-CGCGAACTCCGT-3’ and R sequence of reverse internal standard primer:5’-CACTAGGCGCTCACTGT-3’), internal Standard Probe (ROX-5’-CACCTTCCCCATGGTGTCT-3’ -BHQ2), Taq enzyme,uracil- N-glycosylase (UNG) enzyme, positive control solution (plasmid DNA mixture of *MTHFR 677C* and *MTHFR 677T*) and blank control solution (Tris-HCl buffer (10mM))

Genomic DNA Extraction from Peripheral Blood Samples: Whole anticoagulant blood of 200 μL EDTA was extracted from the subjects’ peripheral veins, and genomic DNA was extracted according to the instructions of the blood genome extraction kit. Tia Namp Blood DNA Kit (DP348, Beijing Tiangen Biochemical Technology Co, Ltd.). Genomic DNA concentration was detected by Nano Drop, and the genomic DNA was stored at -80°C.

Detection of *MTHFR C677T* polymorphism(PCR fluorescence probe): the genomic DNA of the samples to be tested, the positive control and the blank control were added into the reaction tube with the PCR reaction solution at the addition amount of 2μl, and then water was added to form the reaction system of 25μL.Procedures for quantitative fluorescence PCR reaction under the real-time quantitative fluorescence PCR instrument (AB 7500): treatment at 37°C for 10min, pre-denaturation at 95°C for 5min, 40 cycles were performed according to the following procedure: 95°C for 15s, 60°C for 1min.

*MTHFR* genotyping: According to the amplification curve, the appropriate baseline (starting set at 3, ending set at 15) and fluorescence threshold were determined to obtain Ct values of different ROX channels after PCR amplification. Then, according to the Ct value of ROX channel, genotypes can be distinguished as follows: wild-type(*C/C*): FAM channel acuities were 36 Ct values, VIC channel Ct value >36 or without Ct value; heterozygous mutant (*C/T*): FAM channel Ct value ≤36, VIC channel Ct value ≤36; homozygous mutations (*T/T*): FAM channel Ct value > 36 or no Ct, VIC channel Ct value does not exceed 36.

UF diagnosed by BU was obtained by the BU examination system and diagnosed by the clinical obtained by the first page of our hospital’s medical records. Specific clinical characteristics (such as age, height, weight, BMI, number of pregnancies, number of births, size and number of UF) were obtained by the examination system, outpatient and inpatient medical records system. And the height and weight measurements was taken by the health care provider in our hospital. Considering the age, BMI, and gestational history may have an effect on UF, so will be discrete as classification variables for analysis.

### Statistical Analysis

Epidada software was used to establish a database, and statistical software SPSS 25.0 was used to analyze and process the data. The counting data were expressed by mean ± SD (Standard deviation), and the composition ratio expressed the measurement data. The allele frequencies were calculated by the gene frequency counting method, and H-W equilibrium was tested by goodness-fit chi-square test. Genotype and gene frequency were compared between the two groups by the chi-square test, and the relative risk was estimated by the odd rate(OR) value and its 95% confidence interval(CI). A bilateral p value <0.05 was considered statistically significant.

## Results

### Study Characteristics

In this study, a total of 2411 women were detected for peripheral blood *MTHFR C677T* polymorphism and 226(9.73%) women who had been clinically and ultrasonically diagnosed with UF. The mean age when the UF was first detected was (30.9 ± 4.37) years. Relevant demographic characteristics (such as age, height, weight, BMI, parity and gravidity) and UF (size and number of fibroids) in the case and control groups were shown in **Table 1**. There were no statistically significant differences in height, weight, BMI, parity and gravidity, frequency of UF, or distribution of *MTHFR C677T* polymorphism between the case and control groups (p>0.05).In terms of age, older maternal age women were more likely to develop UF(p<0.05). The size and number of UF correlated with *MTHFR C677T* polymorphism (p<0.05).

**Table 1:** General characteristics of study subjects.

Variable	group		P value	Genotype		P value
	Controls n=2185	Cases n=226		*C/C* n=1072	*C/T+T/T* n=1339	
Age(y,mean ± SD)	29.83 ± 4.25	32.56 ± 4.73	0.00	29.77 ± 4.36	30.34 ± 4.37	0.75
<35	1863(92.3)	156(7.7)	0.00	908(45.0)	1111(55.0)	0.25
≥35	322(82.1)	70(17.9)		164(41.8)	228(58.2)	
Height(cm,mean ± SD)	159.84 ± 5.35	159.64 ± 4.78	0.69	159.65 ± 4.87	159.96 ± 5.62	0.06
weight(kg,mean ± SD)	54.60 ± 9.20	55.45 ± 7.96	0.53	54.22 ± 8.93	55.05 ± 9.20	0.06
BMI(kg/m^2^,mean ± SD)	21.41 ± 4.48	21.74 ± 2.87	0.44	21.28 ± 3.39	21.58 ± 4.99	0.07
<18.5	326(92.4)	27(7.6)	0.28	159(45.0)	194(55.0)	0.13
18.5-25	1610(90.6)	167(9.4)		804(45.2)	973(54.8)	
≥25	249(88.6)	32(11.4)		109(38.8)	172(61.2)	
Gravidity(n,mean ± SD)	1.94 ± 1.07	2.08 ± 1.23	0.27	1.92 ± 1.09	1.99 ± 1.09	0.83
1	923(90.3)	99(9.7)	0.66	471(46.1)	551(53.9)	0.17
≥2	1261(90.9)	127(9.1)		601(43.3)	787(56.7)	
Parity(n,mean ± SD)	1.43 ± 0.58	1.42 ± 0.62	0.23	1.43 ± 0.57	1.44 ± 0.59	0.40
unipara	1258(90.4)	137(9.6)	0.60	630(44.3)	792(55.7)	0.85
pluripara	900(91.0)	89(9.0)		442(44.7)	547(55.3)	
size(mm,mean ± SD)	–	–		22.12 ± 9.03	29.33 ± 10.40	0.02
number						<0.05
single	–	–		71	123	
multiple	–	–		12	20	

BMI, Body Mass Index; SD, Standard deviation.

### Correlation Analysis Between UF and *MTHFR C677T* Polymorphism

The incidence of UF was 9.73%(226/2411), which was correlated with *MTHFR C677T* polymorphism (p<0.05). The allele distribution in the case group and the control group was equal to the H-W equilibrium(p>0.05). The incidence of UF for mutations group(*C/T+T/T*)(12.0%,143/1196) is higher than wild-type(*C/C*)(8.4%,83/989). Regression analysis showed that the mutant group (*C/T+T/T*) had a higher risk of UF than the wild-type group(*C/C*)(OR,1.43;OR95%CI,1.07-1.89). After adjusting for age, height, weight, BMI, parity, and gravidity, heterozygous mutant(*C/T*) incidence was still higher than wild-type(*C/C*) (aOR,1.41;aOR95%CI,1.41-1.91) (**Table 2**).

**Table 2:** Correlation analysis between UF and *MTHFR C677T* polymorphism.

MTHFR	Controls	Cases	χ^2^ value	P value	OR95%CI	
	n	n			Unadjusted	adjusted
Allele			3.03	0.08		
*C*	2876	279			1	–
*T*	1494	173			0.84(0.69-1.02)	–
Genotype			7.25	0.03		
*C/C*	989	83			1	
*C/T*	898	113			1.50(1.11-2.02)*	1.41(1.04-1.91)*
*T/T*	298	30			1.20(0.78-1.86)	1.01(0.64-1.58)
*C/T+T/T*	1196	143	6.05	0.01	1.43(1.07-1.89)*	1.31(0.98-1.75)

*P value <0.05, the different was considered statistically significant. OR, odd rate; CI, confidence interval.

### The Predictive Value of *MTHFR C677T* Polymorphism in the Diagnosis of UF

The positive predictive values(PPV) of the allele and gene mutation for UF were 10.4%[173/(1494 + 173)] and 10.7%[143/(143 + 1196)], and the negative predictive values(NPV) were 91.2% [2876/(2876 + 279)] and 93.3% [989/(989 + 83)]. The allele sensitivity was 38.3%[173/(173 + 279)], and the specificity was 65.8%[2876/(2876 + 1494)]. Mutation genotype of sensitivity was 63.3% [143/(83 + 143)], and the specificity was 45.3% [989/(989 + 1196)].

### Correlation Analysis Between *MTHFR C677T* Polymorphism and the Size and Number in the Case Group

**Table 3** showed that the incidence of single UF in the case group was 84.51%(191/226), and the incidence of multiple UF was 15.49%(35/226). The number of UF and the distribution difference of *MTHFR C677T* polymorphism were statistically significant (p<0.05) in the mutant group (*C/T+T/T*). The probability of occurrence of a single UF was relatively high (85.3%). However, there were no significant differences between *MTHFR C677T* polymorphism and the size distribution of UF(p>0.05).

**Table 3:** Correlation analysis between *MTHFR C677T* polymorphism and the size and number in the case group.

	Single n (%)	Multiple n (%)	P value	OR95%CI	≤40mm n (%)	>40mm n (%)	P value	OR95%CI
*MTHFR*			0.04				0.54	
*C/C*	69(83.1)	14(16.9)		1	64(77.1)	19(22.9)		1
*C/T*	101(89.4)	12(10.6)		0.59(0.26-1.34)	90(79.6)	23(20.4)		0.86(0.43-0.71)
*T/T*	21(70.0)	9(30.0)		2.11(0.80-5.57)	26(86.7)	4(13.3)		0.52(0.16-1.67)
*C/T+T/T*	122(85.3)	21(14.7)	0.66	0.20(0.41-1.77)	116(81.1)	27(18.9)	0.47	0.30(0.41-1.52)

OR, odd rate; CI, confidence interval.

### The Influence of Different Demographic Characteristics on the Incidence of UF in the Case Group

In the case group, the distribution between BMI, age, parity and gravidity, the size and number of UF and *MTHFR C677T* polymorphism was shown in **Table 4**. There was no correlation between them. But age was associated with the number of UF, and older maternal age were more likely to have multiple UF (p<0.05).

**Table 4:** The influence of different demographic characteristics on the incidence of UF in the case group (n=226).

Variable	number		P value	size		P value	Genotype		P value
	Single n=191	Multiple n=35		≤40mm n=180	>40mm n=46		*C/C* n=83	*C/T+T/T* n=143	
Age(y)			<0.01			0.79			0.61
<35	139(89.1)	17(10.9)		125(80.1)	31(19.9)		59(37.8)	97(62.2)	
≥35	52(74.3)	18(25.7)		55(78.6)	15(21.4)		24(34.3)	46(65.7)	
BMI(kg/m^2^)			0.40			0.72			0.33
<18.5	25(92.6)	2(7.4)		22(81.5)	5(18.5)		10(37.0)	17(63.0)	
18.5-25	139(83.2)	28(16.8)		131(78.4)	36(21.6)		65(38.9)	102(61.1)	
≥25	27(84.4)	5(15.6)		27(84.4)	5(15.6)		8(25.0)	24(75.0)	
parity			0.65			0.16			0.45
unipara	117(85.4)	20(14.6)		105(76.6)	32(23.4)		53(38.7)	84(61.3)	
multiple	74(83.1)	15(16.9)		75(84.3)	14(15.7)		30(33.7)	59(66.3)	
gravidity			0.22			0.05			0.20
1	87(87.9)	12(12.1)		73(73.7)	26(26.3)		41(41.4)	58(58.6)	
≥2	104(81.9)	23(18.1)		107(84.3)	20(15.7)		42(33.1)	85(66.9)	

BMI, Body Mass Index.

## Discussion

As the most common benign tumor of the female genital tract, UF seriously affects women’s health. Currently, UF etiology has been reported that it is related to genetic factors, fibrosis, OS, and hormone levels. Nevertheless, the specific etiology of that is still unclear. For the first time, our study found that UF incidence was related to *MTHFR C677T* polymorphism, but was not related to the size and number of UF, which may be involved in the occurrence of UF in some way.

Previous studies have shown that the genetic alteration and epigenetic mechanism of leiomyoma are involved in UF occurrence, but the specific mechanism is unclear (1). Maekawa and Tamura et al. found the X chromosome’s abnormal DNA hypomethylation in UF through whole genome DNA methylation analysis (17). Sato found that the UF samples had the hypomethylation of TSPYL2. And the expression of some genes decreased related to X chromosome inactivation (18). Their study suggests that a specific UF type may be associated with abnormal DNA hypomethylation of chromosomes. As a key enzyme in folate metabolism, *MTHFR* is involved in DNA synthesis, modification and methylation (19). The gene mutation may lead to the decrease of related to DNA methylation, which leads to related chromosomal abnormalities and contributes to UF occurrence. A retrospective study of UF showed significant genomic heterogeneity in leiomyoma lesions and identified gene mutations associated with intercellular interaction and extracellular matrix remodeling (20). However, there is no report about the relation of *MTHFR C677T* mutation and UF. Moreover, whether it involves in UF occurrence is unclear. *MTHFR* gene detection in UF samples should further explore.

Fibrosis is a pathological feature of many chronic inflammatory diseases caused by the accumulation of excessive ECM (7). The ECM of UF is mainly composed of collagen, fibronectin and proteoglycan, 50% more than the corresponding myometrium, and is generally considered as a fibrosis disease (21). An animal study showed that FA supplementation significantly reduced blood sugar levels and Hcy levels of heart tissue in mice, thereby reducing myocardial fibrosis and reversing cardiac dysfunction caused by a high-fat diet (9). Another clinical study showed a higher frequency of vitamin B_12_ and FA deficiency in patients with oral submucosal fibrosis (22). These findings suggest that low FA levels are associated with fibrosis and may promote the formation of fibrosis. Recent studies have shown that ECM’s deposition, cell proliferation, and angiogenesis are critical cellular events associated with leiomyoma growth (7). In this study, it found that the incidence of UF in the mutant group (*CT+TT*) was higher than that in the wild type (*C/C*), and heterozygous mutation (*C/T*) was still a risk factor for UF after the correction of mixed ligation factors. Moreover, the sensitivity of genotype mutation to the occurrence prediction of UF is as high as 63.3%. BU still has a certain rate of missed diagnosis of UF, and its combination with BU is helpful for the diagnosis of UF. But its specificity is low, easy to lead to over-examination. A multi-center randomized control analysis was still needed in clinical practice that will be our future research plan.

Previous studies showed that the overall *677T* allele frequency was 36.9% in China, which exceeds that of many other countries (23), Our study found that the ratio of heterozygous mutations (*C/T*)(1011/2411) was higher than that of homozygous mutations (*T/T*)(328/2411). As a key enzyme in folate metabolism, *MTHFR* gene mutation may reduce the activity of folate metabolizing enzymes and increase the concentration of plasma Hcy, leading to oxidative stress and promoting the deposition of extracellular matrix in fibroids. Therefore, the heterozygous mutations (*C/T*) may give the most significant phenotype. There is no relevant research, which may need to be further proved by basic experiments.

FA is an antioxidant that reduces the production of reactive oxygen species. Previous studies have shown that FA supplementation can reduce cardiac dysfunction caused by the OS during ischemia (24). Fibroid cells are characterized by a unique NOX profile, which was the primary source of superoxide and subsequent OS to promote severe prooxidant states (25). And NOX derivative of ROS is SMC proliferation, a vital part of the signal transduction pathways (8). It suggests that OS may be an essential factor in UF formation. The mutation of the *MTHFR C677T* gene leads to the disorder of folate metabolism, the decrease of antioxidant capacity, the increase of uterine related ROS, and the proliferation of uterine smooth muscle cells, thus leading to the formation of UF. Besides, this study found no statistically significant difference between *MTHFR C677T* polymorphism and the size and number of UF, which may only be involved in UF formation. Studies have shown that estrogen promotes UF proliferation by activating fibroblasts, so its size may be correlated with hormone levels (10, 26), but not with *MTHFR C677T* polymorphism. How the gene mutation regulates the signaling pathway of the OS and thus promotes UF occurrence has not been studied yet, which needs to explore later further.

Recently, some emerging ideas have suggested that UF is the result of the chronic active inflammatory immune system, and their occurrence may be related to inflammatory disorders. As mediators of sex steroids, relevant inflammatory factors are involved in the formation and proliferation of UF, fibrosis, and angiogenesis (9). Studies have shown that hyperhomocysteine unregulated the protein expression of mesenteric VEGF. Moreover, phosphorylated-endothelial nitric oxide synthase (P-ENOS) and FA treatment can reverse these effects (16). Studies have shown differential expression of various growth factors involved in angiogenesis in leiomyomas, such as VEGF, basic fibroblast growth factor, activin A and TGF-β, Etc. *MTHFR C677T *polymorphism was associated with increasing Hcy levels (8). The incidence of UF was higher in the homozygous mutation(*C/T*) (**Table 3**). Whether the *MTHFR C677T* polymorphism leads to folate metabolism disorder which in turn leads to increased Hcy levels, promotes the secretion of VEGF, and is involved in the formation of UF. A case-control study proved that eating fruits and green vegetables had a protective effect on UF occurrence (27).

**Advantages and disadvantages:** Our study found that *MTHFR C677T* polymorphism was associated with UF incidence for the first time and was accorded with H-W balance in the case and control groups. It causes mutations, maybe by reducing DNA methylation, promoting UF fibrosis, promote the secretion of OS and abnormal growth factor mediating the happening of the UF, and provide clues for the mechanism of UF. However, this study focused on the relationship between *MTHFR C677T* polymorphism and UF. FA, Hcy, estrogen, progesterone, and related receptors were not included in the analysis, so its promoting effect on UF occurrence could not be excluded. Meanwhile, this study’s number of case groups was still small, and multi-center randomized control analysis was still needed. In the meantime, in clinical practice, the cost of detecting and obtaining mRNA gene expression value is high, and some patients cannot afford the related costs, leading to the delay of diagnosis and treatment of related diseases and adverse outcomes. The same is true for other diseases or tumors. The detecting of mRNA gene expression value has not been widely used in clinical practice.

Prior research has not yet found that the correlation of *MTHFR C677T* polymorphism and uterine fibroids. Here, we observed through PCR-RFLP that the *MTHFR C677T* polymorphism is associated with the onset of UF. A study in the future, we will further test the mutation of *MTHFR C677T* in the myometrium tissue and compare to the difference of peripheral blood and local tissue and the case and control tissue, and further explore the mechanism of *MTHFR C677T* polymorphism participate in forming UF.

In conclusion, The results show that *MTHFR C677T* polymorphism was associated with the occurrence of UF. A broader assessment of this association is necessary to provide a reference for exploring UF’s pathogenesis and address additional genetic variation in FA metabolism pathways. The report suggests that using the larger study populations with prospective sampling in the future to minimize other factors’ opportunities and influence on possible survival advantages.

## Data Availability Statement

The data analyzed in this study is subject to the following licenses/restrictions: The data can be made available by the corresponding author upon reasonable request. Requests to access these datasets should be directed to Jiahui Shen,18718804918@163.com.

## Authors Contributions

QY, JS, and YJ: Conceptualization, Methodology, Software. FW, YC, and HC: Data curation, Writing-Original draft preparation. JS, LC, and YC: Visualization, Investigation. QY and XZ: Supervision. HC, LC, and XZ: Software, Validation: QY, JS, and YJ: Writing-Reviewing and Editing. The United laboratory of SYSU The Fifth Affiliated Hospital-BGI provides the Detection Report of *MTHFR C677T* polymorphism and database. All authors contributed to the article and approved the submitted version.

## Conflict of Interest

The authors declare that the research was conducted in the absence of any commercial or financial relationships that could be construed as a potential conflict of interest.

## Abbreviations

UF, Uterine fibroids; *MTHFR*, Methylenetetrahydrofolate reductase; BU, B-ultrasound; H-W, Hardy-Weinberg; SMC, Smooth muscle cells; ECM, extracellular matrix; OS, oxidative stress; ROS, Reactive oxygen species; FA, Folic acid; Hcy, homocysteine; VEGF, vascular endothelial growth factor; UNG, uracil- N-glycosylase; OR, odd rate; CI, confidence interval; PPV, positive predictive values; NPV, negative predictive values; P-ENOS, phosphorylated-endothelial nitric oxide synthase.
